# Twists of the Gut and Genome: A Case of Intestinal Intussusception Revealing Lynch Syndrome in a Young Adult and Literature Review

**DOI:** 10.7759/cureus.87738

**Published:** 2025-07-11

**Authors:** Imran Khokhar, Eldia Delia, Gisha Mohan, Jason Farrell, Anish Paudel

**Affiliations:** 1 Internal Medicine, Reading Hospital, Tower Health, West Reading, USA; 2 Internal Medicine, Suburban Community Hospital, Norristown, USA; 3 Gastroenterology and Hepatology, Reading Hospital, Tower Health, West Reading, USA

**Keywords:** adenocarcinoma, gastroenterology, ileocolic, intussusception, lynch syndrome, surgery, young woman

## Abstract

Adult intussusception is a rare clinical entity and often indicates an underlying organic pathology, particularly malignancy. Unlike pediatric cases, adult intussusception necessitates oncologic evaluation and surgical management.

We present a case of a 32-year-old woman with no family history of colorectal cancer who presented with a three-month history of intermittent abdominal pain, which had worsened recently. Computed tomography imaging revealed a target-like lesion in the cecum and ascending colon, consistent with ileocolic intussusception. Exploratory laparotomy identified a 10 cm intussuscepted segment with a thickened bowel wall and regional lymphadenopathy. A right hemicolectomy with en-bloc resection and lymphadenectomy was performed. Histopathology confirmed a poorly differentiated tubular adenocarcinoma invading the muscularis propria (T2N0M0) without lymph node involvement. Immunohistochemistry demonstrated loss of MLH1, MSH2, and MSH6, consistent with microsatellite instability-high (MSI-H) phenotype. Genetic testing confirmed Lynch syndrome. The postoperative course was uneventful, and the patient was discharged on postoperative day five without the need for adjuvant chemotherapy.

Ileocolic intussusception in young adults may be the initial presentation of colorectal malignancy, including hereditary cancer syndromes. Timely surgical resection and genetic evaluation are crucial for diagnosis, staging, and long-term management.

## Introduction

Intussusception is most commonly seen in adults as a secondary result of an underlying organic pathology in the gastrointestinal tract, in contrast to the pediatric population, where it is often idiopathic [1-3]. In adults, intussusception typically necessitates a more detailed examination with a focus on oncological investigations and management, rather than immediate reduction of the intussusception itself [2,4,5]. Recently, there has been an observed increase in the incidence of primary malignancies of the gastrointestinal tract among individuals under the age of 50 years [4]. Approximately 10-20% of these younger patients may present with symptoms related to intussusception, warranting a high index of suspicion and prompt evaluation following oncological guidelines [6-8]. We describe a case of ileocolic intussusception revealing a poorly differentiated adenocarcinoma in a young woman, successfully managed through surgical intervention [8,9].

This article was previously presented as a poster at the American College of Gastroenterology Annual Conference meeting on November 2, 2021. Informed written consent was obtained from the patient for the open-access publication of this case report.

## Case presentation

A 32-year-old woman with no significant past medical history, no family history of colon adenocarcinoma or any other gastrointestinal malignancies, presented to our emergency department with a three-month history of intermittent abdominal pain accompanied by nausea and vomiting, with the current acute episode of generalized abdominal pain starting four days before presentation. The pain was characterized as feeling like "gut twisting," with severe 10/10 intensity, and generalized abdominal pain. Her first episode of similar type of symptoms was three months ago, in the middle of the night, and resolved in a few minutes without intervention. In the last three to four days, her pain became more frequent, and episodes lasted throughout the day. The pain was initially non-radiating, but started to radiate to her back and right groin eventually. No triggering, worsening, and relieving factors were identified. Her previous pain episodes were used to get better after bowel movements. She tried ibuprofen without any improvement. The abdominal pain was associated with bloating, nausea, non-projectile non-bloody vomiting, and non-bloody diarrhea. She did not report any chest pain, shortness of breath, dysuria, hematuria, recent weight changes, or appetite changes. She denied any personal or family history of ulcerative colitis, Crohn's disease, celiac disease, ovarian torsion, or ovarian cysts. She denied any recent flu or COVID-19 and other vaccines. She did not report any recent upper respiratory infections (URIs) or gastrointestinal viral infections. She did not take any medications and was not allergic to any medications or foods. She denied any current alcohol, smoking, illicit drugs, or recent surgical procedures. She was a former smoker. She reported being up to date on all her vaccines. She usually had irregular menstruation periods, usually light, and lasted three to five days. Her last period was a week ago. She has never been pregnant, and her last sexual encounter was five years ago. No history of STDs was noted. Upon presentation to the emergency room, she had normal vitals. Physical exam was normal, except mild nonspecific tenderness in the right lower quadrant, and the rest of the abdomen was soft, non-tender, non-distended, and had active bowel sounds. No masses were palpated, and no guarding or rebound tenderness was appreciated. Murphy's sign was negative, along with McBurney's or Rovsing's sign. The patient's laboratory findings were within or near normal limits across most parameters (Table 1).

**Table 1:** Summary of laboratory investigations.

Laboratory test	Patient value	Reference range	Interpretation
White blood cell (WBC) count	10.3 ×10³/μL	4.0–11.0 ×10³/μL	↔ Within normal range
Hemoglobin (Hb)	12.6 g/dL	12.0–15.5 g/dL (female)	↔ Within normal range
Platelet count	103 ×10³/μL	150–400 ×10³/μL	↓ Decreased
Sodium (Na⁺)	142 mmol/L	135–145 mmol/L	↔ Within normal range
Potassium (K⁺)	3.9 mmol/L	3.5–5.0 mmol/L	↔ Within normal range
Chloride (Cl⁻)	103 mmol/L	96–106 mmol/L	↔ Within normal range
Blood urea nitrogen (BUN)	7 mg/dL	7–20 mg/dL	↔ Lower end of normal
Creatinine	0.7 mg/dL	0.6–1.3 mg/dL	↔ Within normal range
Glucose	119 mg/dL	70–99 mg/dL (fasting)	↑ Mildly elevated
Calcium (Ca²⁺)	9.4 mg/dL	8.5–10.5 mg/dL	↔ Within normal range
Total bilirubin	0.3 mg/dL	0.1–1.2 mg/dL	↔ Within normal range
Aspartate aminotransferase (AST)	16 U/L	10–40 U/L	↔ Within normal range
Alanine aminotransferase (ALT)	23 U/L	7–56 U/L	↔ Within normal range
Alkaline phosphatase (ALP)	43 U/L	44–147 U/L	↓ Slightly decreased
Lipase	81 U/L	0–160 U/L	↔ Within normal range
Lactic acid (lactate)	0.6 mmol/L	0.5–2.2 mmol/L	↔ Within normal range

**Figure 1:**
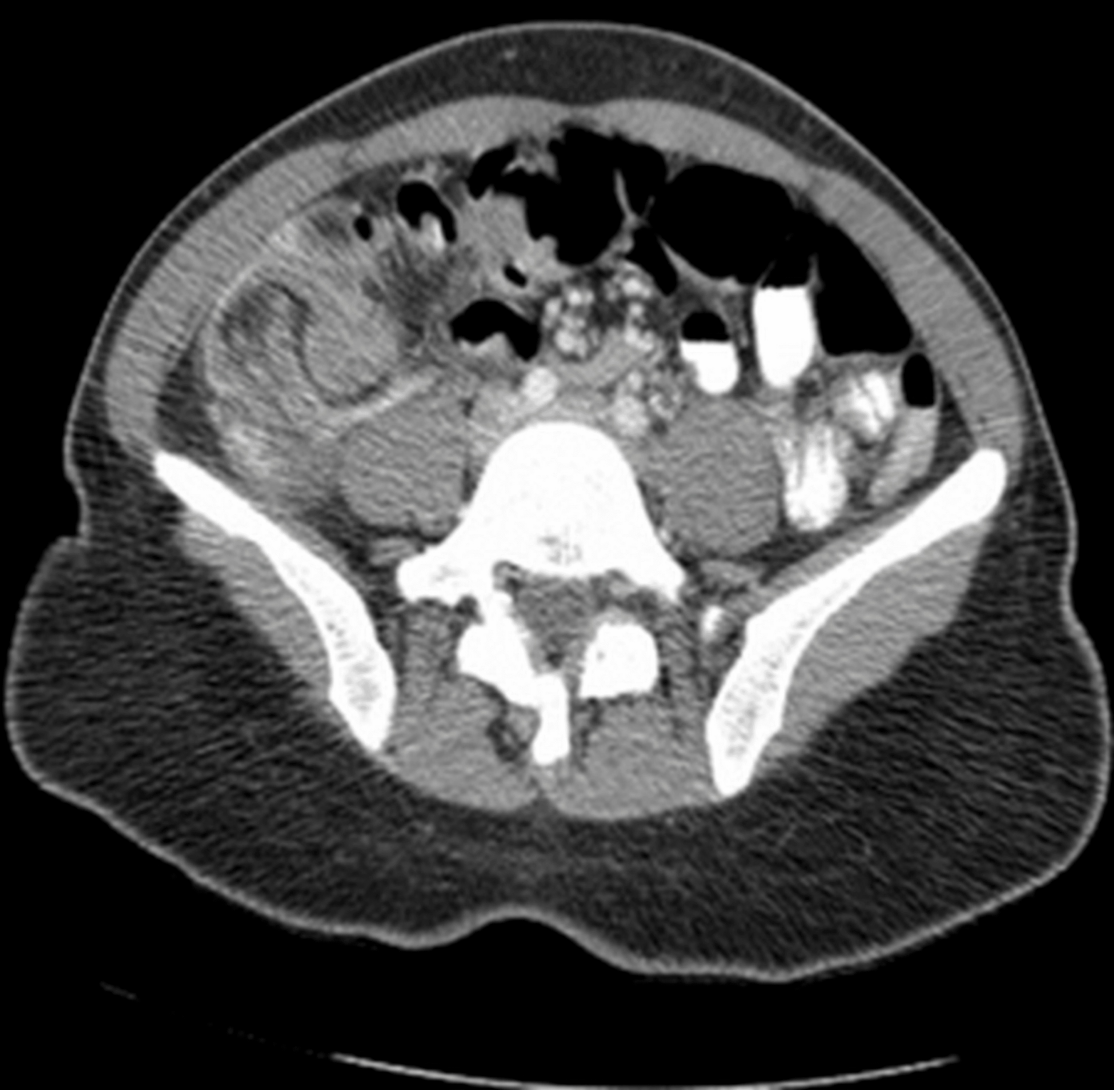
Computed tomography scan of the abdomen (axial view) showing the intussusception at the ileocecal junction.

**Figure 2:**
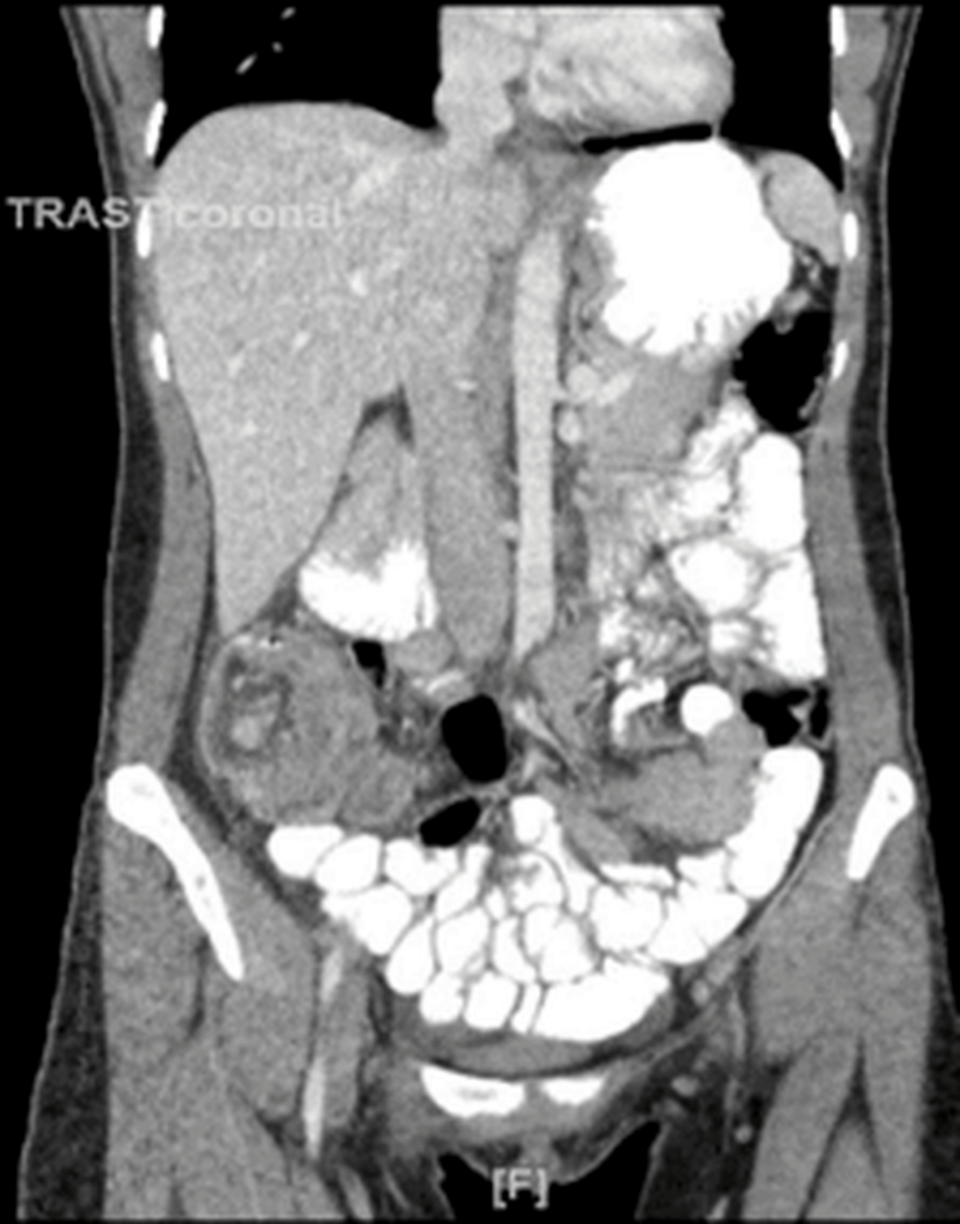
Coronal view of the CT scan of the abdomen with contrast showing non-distended small bowel loops and ileocecal intussusception.

A computed tomography (CT) of the abdomen and pelvis with intravenous contrast was performed due to abdominal pain. CT scan images showed inflammatory changes in the right lower quadrant and an appearance consistent with ileocolic intussusception measuring approximately 10 cm in length (Figures 1, 2).

There was inflammatory fat stranding and mild free fluid at the site of the intussusception. There were several mildly enlarged right lower quadrant mesenteric lymph nodes, some of which seemed to lie within the intussusception. There was a small amount of oral contrast within the rectum. The ascending colon and transverse colon appeared relatively decompressed. The oral contrast in the distal large bowel excluded complete obstruction. There were no dilated loops of small bowel to suggest small bowel obstruction (SBO).

The gastroenterology team was consulted, and they recommended against colonoscopy due to the presence of abdominal pain, intussusception in the right colon, and likely stool burden. Tap water enema was not tried, given the proximal presence of intussusception. The patient failed conservative management. The surgery team was consulted due to persistent abdominal pain and tenderness in the right lower quadrant and failure of conservative management.

The patient was planned for exploratory laparotomy. Intra-operatively, a 10-cm ileocolic intussusception was found with thickening of the colonic wall and slight proximal intestinal dilation. Multiple lymphadenopathies in the region of intussusception were observed. Right hemicolectomy was performed following strict oncologic principles with "en-bloc resection" and lymphadenectomy given the risk of an underlying malignancy. Considering this risk, the previous reduction of the invaginated segments was not attempted, and primary extracorporeal anastomosis was performed using manual sutures.

**Figure 3:**
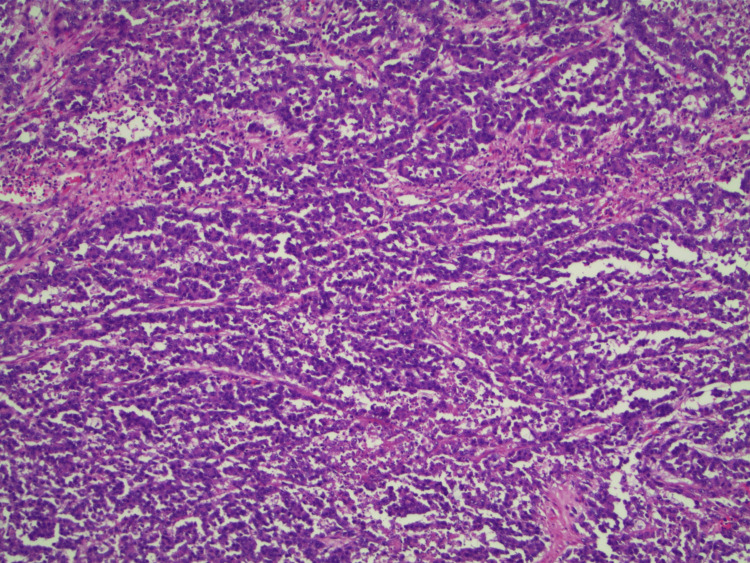
Histopathological image of the adenocarcinoma showing infiltrative, irregular glandular structures lined by pleomorphic, hyperchromatic epithelial cells with loss of polarity and prominent nucleoli. Tumor glands are embedded in a desmoplastic stroma with scattered inflammatory infiltrates.

**Figure 4:**
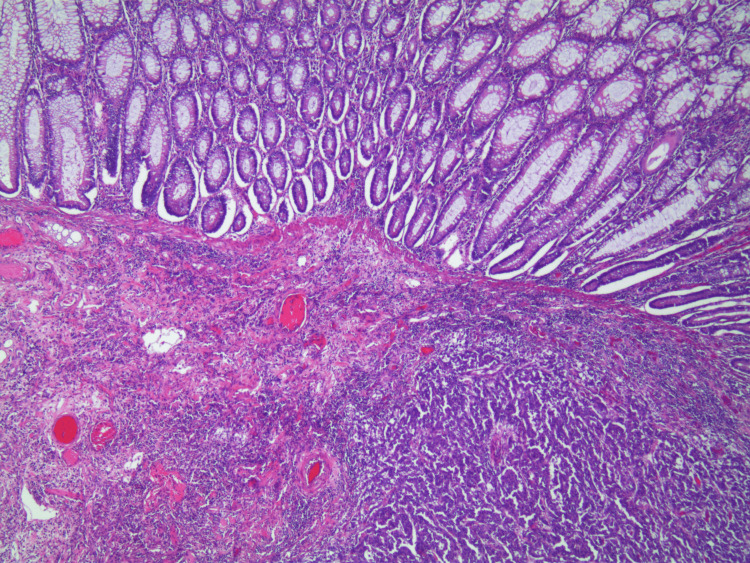
Histopathological image of the adenocarcinoma showing irregular infiltrative malignant glands invading through the muscularis mucosa into the submucosa. The tumor displays nuclear pleomorphism, loss of polarity, and is surrounded by desmoplastic stroma and inflammatory infiltrate, adjacent to preserved colonic mucosa.

Macroscopic examination of the resected specimen revealed a tumor mass of the cecal wall. The lesion originated in the cecum in the vicinity of the appendiceal orifice; however, the appendix was uninvolved. The histological analysis (Figures 3, 4) identified a poorly differentiated tubular adenocarcinoma invading muscularis propria (T2) without permeation of the lymphatic or venous capillaries.

No lymphatic metastasis was seen in the 23 nodes removed. Surgical margins were free of malignancy. The postoperative course was uneventful, and the patient was discharged five days after surgery with outpatient follow-up with the surgery and oncology team.

Postoperative chest, abdomen, and pelvis CT scans were normal. Therefore, the tumor was classified as stage I (T2N0M0). There was a loss of MLH1, MLH2, and MSH6 protein expression on immunohistochemistry findings, reflecting a microsatellite instability phenotype, CDX-2 positivity consistent with colorectal adenocarcinoma, and Lynch syndrome was diagnosed based on high-frequency molecular satellite instability (MSI-H) on genetic testing. The patient was followed up without adjuvant chemotherapy.

## Discussion

Paul Barbette described intussusception for the first time in 1674, while Scottish surgeon James Hunter coined this term in 1793 [1]. Intussusception is the telescoping or invagination of one segment of the bowel into an adjacent bowel segment, leading to edema, venous congestion, and reduction in blood supply, eventually causing ischemia [1,2]. This can occur anywhere in the small or large bowel. Based on the position of the lead point (LP), intussusception can be entero-enteric (small bowel only), colo-colonic (large bowel only), ileocecal (ileocecal valve being LP), and ileocolic (terminal ileum prolapsing into the ascending colon) [1]. Intussusception is common in childhood between six to 18 months. It is typically idiopathic and benign, with ileocolic as the most common presentation [2]. Adult intussusceptions are rare, with two to three per million cases worldwide annually, and they most commonly occur in the small bowel, while 10% of the cases take place in the stomach or near the surgical stoma site [3]. Its mean age of presentation is 50 years, without any gender predominance. In around 70-90% of the cases of adult intestinal intussusception, an identifiable pathology is reported [3].

Colorectal cancer predominantly affects males, and its incidence increases with age. Despite this, Bailey et al. highlighted an increasing incidence in younger patients, between 20 and 34 years of age, which could increase by 90% for colon cancer and 124.2% for rectal cancer by 2030 [4]. Obesity, diets rich in red meat and processed foods, and the presence of inflammatory intestinal diseases are considered risk factors for colorectal cancer [4]. Approximately 30% of colorectal cancers are associated with genetic factors, and 5% are attributed to syndromes known to cause hereditary colorectal cancers. Among them are mutations in the DNA repair system, like Lynch syndrome, mutations in the APC gene like familial adenomatous polyposis, and some uncommon syndromes like Peutz-Jegher's syndrome and type X colorectal cancer [5].

In patients like ours, presenting with Lynch syndrome without any previous family history, it is difficult to diagnose asymptomatic patients before 50 years of age. Beyond 50 years, colonoscopy screening and subsequent follow-ups increase the chances of diagnosing colonic cancer. A review of the literature showed very few case reports describing adult intestinal intussusception attributable to tumors of the colon seen before 50 years of age. This syndrome is responsible for 2-4% of all colorectal cancers [5]. Polyps and colon cancer arise at a young age and, compared to sporadic neoplasms, the lesions appear at a more proximal location [5]. Based on histological findings, the masses are usually poorly differentiated, are mucinous, and have increased infiltrating lymphocytes in the tumor [5]. de Mesquita et al. reported symptomatic adult intussusception in a 44-year-old due to colonic adenocarcinoma (mutation in the MUTYH gene) [6]. Asokan et al. reported a 36-year-old patient with intussusception due to a tumor at the recto-sigmoid location but without having a genetic syndrome [7]. Inada et al. reported intussusception in a 24-year-old due to rectal adenocarcinoma. Analysis for the genetic syndrome was only carried out for Lynch syndrome (patient tested negative); however, other genetic mutations were not tested for; thus, a genetic factor was not ruled out [8]. Green et al. reported ileocolic intussusception in a 42-year-old presenting with acute abdominal pain, where the patient was found to have a low-grade appendiceal mucinous neoplasm on surgical pathology acting as the lead point for the intussusception to take place [9]. Taher et al. reported colo-colonic intussusception in a 28-year-old presenting with diarrhea and abdominal pain who was found to have tubule-villous adenoma on surgical pathology, causing a colo-colonic intussusception in the descending colon; however, no genetic testing was performed (Table 2) [10].

**Table 2:** Comparative analysis of studies on adult intussusception and related malignancies. SEER: Surveillance, Epidemiology, and End Results; CRC: colorectal cancer; MSI-H: microsatellite instability-high.

Author (year)	Study type	Population/focus	Key findings	Relevance to the current case
Honjo et al. (2015) [1]	Retrospective review	44 adult intussusception cases	65.9% had identifiable lead points; 32% due to malignancy	Emphasizes the need for oncologic surgical resection in adults
Marsicovetere et al. (2017) [2]	Review article	Literature overview	Adult intussusception is rare; 70–90% have a pathologic lead point	Supports surgical resection over reduction due to high malignancy risk
Behrooz & Cleasby (2018) [3]	Case report with review	Gastrogastric intussusception	Rare, variable presentations in adults	Highlights diagnostic variability and the need for CT imaging
Bailey et al. (2015) [4]	Population study	SEER data, age <50 vs. >50	Rising CRC incidence in the under-50 age group	Supports increased surveillance for malignancy in young adults
Jasperson et al. (2010) [5]	Review	Hereditary colon cancer syndromes	Outlines Lynch syndrome criteria and management	Provides background for genetic diagnosis in this case
de Mesquita et al. (2019) [6]	Case report	MUTYH-associated polyposis with intussusception	CRC revealed through intussusception	Demonstrates the genetic causes of intussusception-related CRC
Asokan & Hollington (2014) [7]	Case report	Recto-sigmoid intussusception with malignancy	Malignant cause in an adult female	Aligns with malignant lead point without prior family history
Inada et al. (2014) [8]	Case report	Rectal adenocarcinoma in a young adult	MSI-H phenotype with intussusception	Mirrors the genetic profile and tumor behavior of the current case
Green et al. (2019) [9]	Case report	Appendiceal mass causing ileocolic intussusception	Non-malignant, rare cause in adults	Supports anatomical location relevance to the current case
Taher et al. (2019) [10]	Case report	Colocolonic intussusception from the descending colon	Rare presentation, surgical resection essential	Demonstrates the surgical principle of en bloc resection
Brill & Lopez (2021) [11]	Textbook chapter	Overview of adult intussusception	Etiology, diagnosis, and treatment are summarized	Foundational reference for case discussion
Kim KH et al. (2014) [12]	Clinical study	41 adult intussusception cases	Identified CT predictors of malignancy (e.g., wall thickening)	Correlates with CT findings in the current case
Kim JW et al. (2018) [13]	Multicenter study	74 patients from university hospitals	Malignant causes in 45.9%; suggests no attempt at reduction	Strongly supports the non-reduction surgical strategy used

Our case demonstrates the importance of genetic testing, meticulous diagnosis, surgical management, and subsequent follow-up. Ileocolic intussusception is a rare presentation of cancer in young adults. Surgical management is imperative in the diagnosis and treatment of this pathology due to the presence of primary or secondary malignant tumors in 20-50% of cases. Oncologic and genetic consultation should be required for a malignant lesion. However, whether intussusception should be reduced before resection remains controversial. This highly depends on the type of intussusception. In some cases, reduction is preferred, while other schools of thought would rather avoid it to eliminate the possibility of malignant dissemination of the malignant cells underlying the pathology [11]. Current evidence still holds dispute regarding the need to do manual reduction and the range of resection. The presence of lead paint is a definitive correlation with malignancy. Enteric intussusception in adults is mostly related to benign etiology, whereas ileocolic and colonic intussusception have a high degree of relation with malignant etiology. Malignant neoplasms constitute 65-70% of intussusception in the large intestine and 30% in the small intestine [11,12]. Ultrasonography and computed tomography are beneficial diagnostic tools in these cases [12]. Age > 60 years, chronic symptoms > 14 days, and colonic-type intussusception are independent predictive factors for malignancy [13]. A study by Kim et al. showed that in patients with chronic symptoms or colonic intussusception, en bloc resection should be considered, but the reduction before carrying out a resection may be considered in small bowel tumors, the majority of which are benign [13].

## Conclusions

In conclusion, intestinal intussusception may be classified based on etiology or location. In this paper, we focused on malignant etiologies acting as a lead point for intussusception to occur. Colonic intussusception is significant for a malignant process, and a careful approach in treatment is needed. Small bowel intussusception is more benign, but careful assessment is needed to analyze the benefit of resection versus reduction. Surgical management is imperative in the diagnosis and treatment of this pathology. Oncologic and genetic consultation should be required for a malignant lesion.

Ileocecal mass/intussusception could be the presentation of Lynch syndrome in a young adult without a significant family history. Surgical resection, histology, and immunohistochemistry with genetic analysis are crucial. Lynch syndrome in young women has a long way to go regarding follow-up and screening, so as not to miss other malignancies in different systems.
